# Reviewed article: Silva FO, Carvalho AL, Schnepper GGM, Marchesi LD, Leite IR, Endo LV, Lima CDVS, Massa KHC. Association between diagnosis of depression and health servisse coverage: a multilevel analysis of the National Health Survey, Brazil, 2019. Epidemiol Serv Saude. 2026:35;e20250666

**DOI:** 10.1590/S2237-96222026v35e20250666.b

**Published:** 2026-02-27

**Authors:** Max Moura de Oliveira

**Affiliations:** 1 Universidade Federal de Goiás, Goiânia, GO, Brazil

## Peer review B

## Questionnaire

Does the manuscript contain new and significant information to justify publication? Yes

Does the Abstract (Summary) clearly and accurately describe the content of the article? Yes

Is the problem significant and concisely stated? No

Are the methods described comprehensively? No

Are the interpretations and conclusions justified by the results? Yes

Is adequate reference made to other work in the field? Yes

## Manuscript Structure

Length of article is: Adequate

Number of tables is: Too few

Number of figures is: Adequate

Please state any conflict(s) of interest that you have in relation to the review of this paper. None

Interest: Excellent

Quality: Good

Originality: Excellent

Overall: Good

Recommendation: Major Revision

## Comments

Prezados(as) autores(as),

Agradeço a oportunidade de revisar o manuscrito “Associação entre depressão e cobertura por serviços de saúde no Brasil: Pesquisa Nacional de Saúde 2019”. O tema abordado é de grande relevância para a saúde pública no Brasil, dado o impacto da depressão e a importância do acesso a serviços de saúde. O uso de dados de uma pesquisa nacional como a PNS 2019 confere um grande potencial ao estudo.

Seguem comentários e sugestões para aprimoramento do trabalho:

Título:

O título é claro e conciso. No entanto, sugere-se uma revisão para refletir os resultados observados, bem como a metodologia utilizada, uma vez que a perspectiva multinível ainda é pouco explorada no âmbito dos inquéritos brasileiros.

Resumo:

Sem observações.

Introdução:

O tema da depressão tem sido bastante explorado, desta forma, poderia ser mais focado nas lacunas que o estudo pretende abordar.

Rever a citação “Segundo o Boletim Epidemiológico do Ministério da Saúde (2), a prevalência de depressão em adultos no Brasil em 2019 foi de aproximadamente 10%, com maior ocorrência em mulheres (15%) comparado aos homens (5%).”, pois não há necessidade de citar o boletim, uma vez que posteriormente é citado um artigo que aborda os mesmos resultados (referência 9).

Explorar mais a justificativa para a perspectiva multinível. Qual a necessidade de conhecer a associação entre depressão e cobertura dos serviços de saúde numa perspectiva multinível?

Apresentar as hipóteses específicas do porquê a associação entre depressão e cobertura dos serviços de saúde deve ser estudada em um nível contextual (unidades federativas). Além disso, explicar como essa abordagem multinível contribuirá para um entendimento mais aprofundado do problema, indo além dos fatores individuais.

A frase “Nesse contexto, o presente estudo tem como objetivo investigar a associação entre a presença de depressão e fatores individuais e ambientais em adultos brasileiros, contribuindo para a compreensão dos determinantes dessa condição e subsidiando estratégias de prevenção e manejo no país” deve ser revista. Ela parece apresentar o objetivo geral de um projeto de pesquisa, e não o objetivo específico deste artigo.

O objetivo específico (“Analisar a associação entre a cobertura dos serviços de saúde e a presença de depressão em adultos brasileiros”) pode ser introduzido de forma mais direta, evitando a repetição. Adicionalmente, rever a redação do objetivo, pois não traduz a análise apresentada no manuscrito.

Métodos:

Sugere-se rever a descrição das variáveis contextuais. Considerando os indicadores utilizados, os mesmos não parecem ser fatores ambientais, e sim fatores de acesso. Além disso, rever a forma de apresentação dos indicadores, por exemplo, NASF e CAPS foram tratados como número de centros/serviços. Por que dessa escolha? Não seria mais adequado apresentar em número relativo, uma vez que a quantidade irá variar com o tamanho da população?

Por que renda per capita foi utilizado no nível contextual, e não no individual, uma vez que propôs trabalhar com características socioeconômicas e a PNS fornece essa informação?

Rever a descrição da análise estatística.

Cálculo da Prevalência e Testes na Tabela 1: Na seção de “Análise Estatística”, não foi explicitamente mencionado como a prevalência de depressão foi calculada para a Tabela 1 e o teste utilizado (aparece somente na tabela).

A Tabela 2 é apresentada como “resultados dos modelos de regressão logística multinível para a presença de depressão em adultos, considerando características individuais”. Contudo, a estrutura da Tabela 2 (“Modelo 1” e “Modelo 2”) parece uma abordagem de regressão hierárquica do que a um modelo multinível que explicaria a variância entre os níveis. A descrição dos modelos na seção de Análise Estatística precisa ser mais clara sobre a diferenciação e o propósito de cada modelo (Modelo 1, 2 e 3 na Tabela 3).

A Tabela 3 apresenta os resultados do nível contextual. Para demonstrar a real contribuição da análise multinível, sugere-se incluir na Tabela 3 (ou em outra seção pertinente da análise estatística) métricas como a Percentual de Mudança da Variância (PCV) ou o Coeficiente de Correlação Intraclasse (ICC).

Resultados:

Os resultados são apresentados de forma clara e organizada, entretanto, o texto dos resultados poderia ser ligeiramente condensado em algumas partes para evitar a repetição excessiva de dados já presentes nas tabelas, focando mais na interpretação dos achados principais.

Discussão:

A discussão dos fatores individuais poderia ser condensada para ter mais espaço para ampliar a discussão a nível contextual. Como mencionado anteriormente, o tema já é bastante explorado. Caso seja interesse explorar mais esses fatores, sugere-se apresentar na Tabela 3 o modelo completo e discutir o efeito do nível contextual no nível individual (os resultados do nível individual foram suprimidos).

A discussão aborda a ideia de que maior cobertura leva a maior detecção, mas poderia aprofundar a nuance entre “maior prevalência real da doença” e “maior prevalência de diagnóstico/detecção”. Embora o estudo transversal não permita inferir causalidade, essa distinção é crucial para a interpretação dos resultados e as implicações políticas. Sugere-se que essa discussão seja mais aprofundada.

Os autores apontam que os achados subsidiam estratégias de prevenção e manejo. A discussão sobre o fortalecimento e expansão da RAPS é boa, mas poderia ser mais específica sobre como os achados do estudo podem guiar gestores em decisões práticas, especialmente para as populações e regiões identificadas com maior ou menor prevalência/detecção, considerando as particularidades da organização dos serviços em cada quartil de cobertura.

